# Deep learning based on multiparametric MRI for differentiating benign from malignant PI-RADS 3 prostate lesions: a dual-center study

**DOI:** 10.1038/s41598-026-56643-x

**Published:** 2026-06-05

**Authors:** He Zhang, Yongfei Zheng, WeiQun Ao, Jingfeng Ding, Binghui Liu, He Huang, GuoHua Zhang, Danjiang Huang

**Affiliations:** 1 Department of Radiology, School of Medicine, Taizhou First People’s Hospital, Taizhou University, Taizhou, Zhejiang Province China; 2 Department of Radiology, Tongde Hospital of Zhejiang Province, Hangzhou, Zhejiang Province China; 3 Putuo People’s Hospital, School of Medicine, Tongji University, Shanghai, China; 4 Department of Pathology, School of Medicine, Taizhou First People’s Hospital, Taizhou University, Taizhou, Zhejiang Province China; 5 Department of Radiology, School of Medicine, Taizhou First People’s Hospital, Taizhou University, No.218, Hengjie RoadHuangyan District, Taizhou City, 318020 Zhejiang Province China

**Keywords:** Prostate cancer, Multiparametric MRI, Deep learning, PI-RADS score 3, Nomogram, Cancer, Computational biology and bioinformatics, Medical research, Oncology

## Abstract

To address the diagnostic challenge of indeterminate PI-RADS 3 prostate lesions, we developed a non-invasive deep learning radiomics model using multiparametric MRI. This dual-center retrospective study included 382 patients. Lesions on T2WI, DWI, and ADC were segmented and standardized. Deep learning features were extracted via ResNet101, and radiomics features were selected using minimum Redundancy Maximum Relevance (mRMR) and Least Absolute Shrinkage and Selection Operator (LASSO). We built and compared three models: clinical, deep-learning radiomics signature (DLRS), and a combined model. The combined model achieved the highest AUCs (0.951, 0.964, 0.925), significantly outperforming the clinical model (*p* < 0.05). The DLRS model also showed high performance (AUCs: 0.946, 0.932, 0.918). The nomogram was well-calibrated, and DCA indicated clinical utility. This mpMRI-based deep learning model accurately differentiates malignant from benign PI-RADS 3 lesions, offering a valuable non-invasive tool to aid clinical decisions and reduce unnecessary biopsies.

**Supplementary Information:**

The online version contains supplementary material available at 10.1038/s41598-026-56643-x.

## Introduction

Prostate cancer (PCa) ranks among the most commonly diagnosed malignancies affecting the male urogenital system worldwide^1,2^. The stage at which prostate cancer is diagnosed significantly influences patient prognosis. Patients identified in early, localized stages typically achieve excellent long-term survival outcomes, with survival rates exceeding 99% at ten years. Conversely, patients diagnosed at advanced or metastatic stages face markedly worse prognoses, with five-year survival rates dropping below 30%^3^. In clinical practice, routine prostate-specific antigen (PSA) screening coupled with multiparametric magnetic resonance imaging (mpMRI) facilitates early cancer detection and stratification of disease risk. Although PSA tests exhibit high sensitivity, their specificity is notably limited, especially when PSA levels range between 4 and 10 ng/ml, where only approximately 26% of biopsies yield positive results^4,5^. mpMRI is commonly used as an auxiliary diagnostic tool due to its crucial role in localizing and characterizing suspicious prostate lesions and guiding biopsies^6^.

The Prostate Imaging Reporting and Data System (PI-RADS) version 2.1 provides critical standardized guidelines for prostate MRI assessment and interpretation, playing a central role in early PCa diagnosis, risk classification, and biopsy guidance^7,8^. Nonetheless, PI-RADS category 3 lesions, termed “equivocal,” pose diagnostic difficulties due to their indistinct benign and malignant characteristics, leading to considerable uncertainty in current clinical evaluations^9^. Previous reports indicate that as many as 88% of biopsies performed on patients with PI-RADS 3 lesions result in benign outcomes^10^, highlighting the challenge in determining their true pathological nature. Consequently, such diagnostic ambiguity increases the potential for clinical overdiagnosis or missed malignancies. Therefore, enhancing diagnostic accuracy for PI-RADS 3 nodules is critical, as precise differentiation between benign and malignant nodules can substantially inform therapeutic strategies and prognostic assessments.

Radiomics converts medical images into quantifiable high-dimensional features reflecting tumor morphology, texture, and heterogeneity, assisting in disease diagnosis and prognosis evaluation^11–14^. Recently, deep learning (DL) approaches, particularly convolutional neural networks (CNNs), have demonstrated significant promise as an advanced subset of machine learning methodologies. DL models inherently extract representative features directly from raw imaging data, effectively overcoming the limitations associated with traditional methods that depend heavily on manual feature extraction, and are particularly adept at analyzing complex and unstructured medical images^15,16^.

Previous studies have attempted to distinguish between benign and malignant PI-RADS prostate 3 lesions using radiomics methods^17,18^. However, some of these studies employed traditional machine learning algorithms or lacked external validation, limiting their generalizability and stability. Additionally, the importance and interpretability of the selected features require improvement. Some prior studies have employed comprehensive slice-by-slice lesion delineation strategies to develop DL models^19^, however, such an approach requires considerable time and labor. To mitigate these limitations, our study employed a single-slice ROI annotation strategy coupled with a deep learning approach, specifically addressing two research objectives: (1) determining whether DL models demonstrate significantly improved performance compared with traditional machine learning techniques, and (2) interpreting feature importance and exploring intrinsic correlations derived from the DL framework.

In response to these limitations, this study established a predictive model employing multiparametric MRI datasets from two separate medical centers, leveraging deep learning methodologies to enhance the differentiation of benign versus malignant PI-RADS 3 prostate nodules. Through rigorous evaluation using internal and external validation cohorts, the robustness, generalizability, and practical applicability of the model were comprehensively verified. Ultimately, this approach aims to provide clinicians with a reliable, non-invasive diagnostic tool to improve preoperative assessments, thereby advancing precision medicine.

## Methods

### Patient characteristics

This retrospective study was reviewed and approved by the ethics committees of both participating institutions. The Medical Ethics Committee of Taizhou First People’s Hospital (Approval No. 2024-KY006-01) explicitly stated in its review evaluation that this study is a retrospective clinical study with potential scientific value and poses no harm to the interests or safety of the subjects; accordingly, the waiver of informed consent and waiver of informed consent signature were formally granted. The Medical Ethics Committee of Putuo District People’s Hospital, Shanghai (Approval No. 2022-07) similarly approved the waiver of informed consent, based on the retrospective nature of the study and the fact that all patient data were fully anonymized and de-identified prior to analysis. All procedures were conducted in accordance with the ethical standards of the Declaration of Helsinki.

**Fig. 1:**
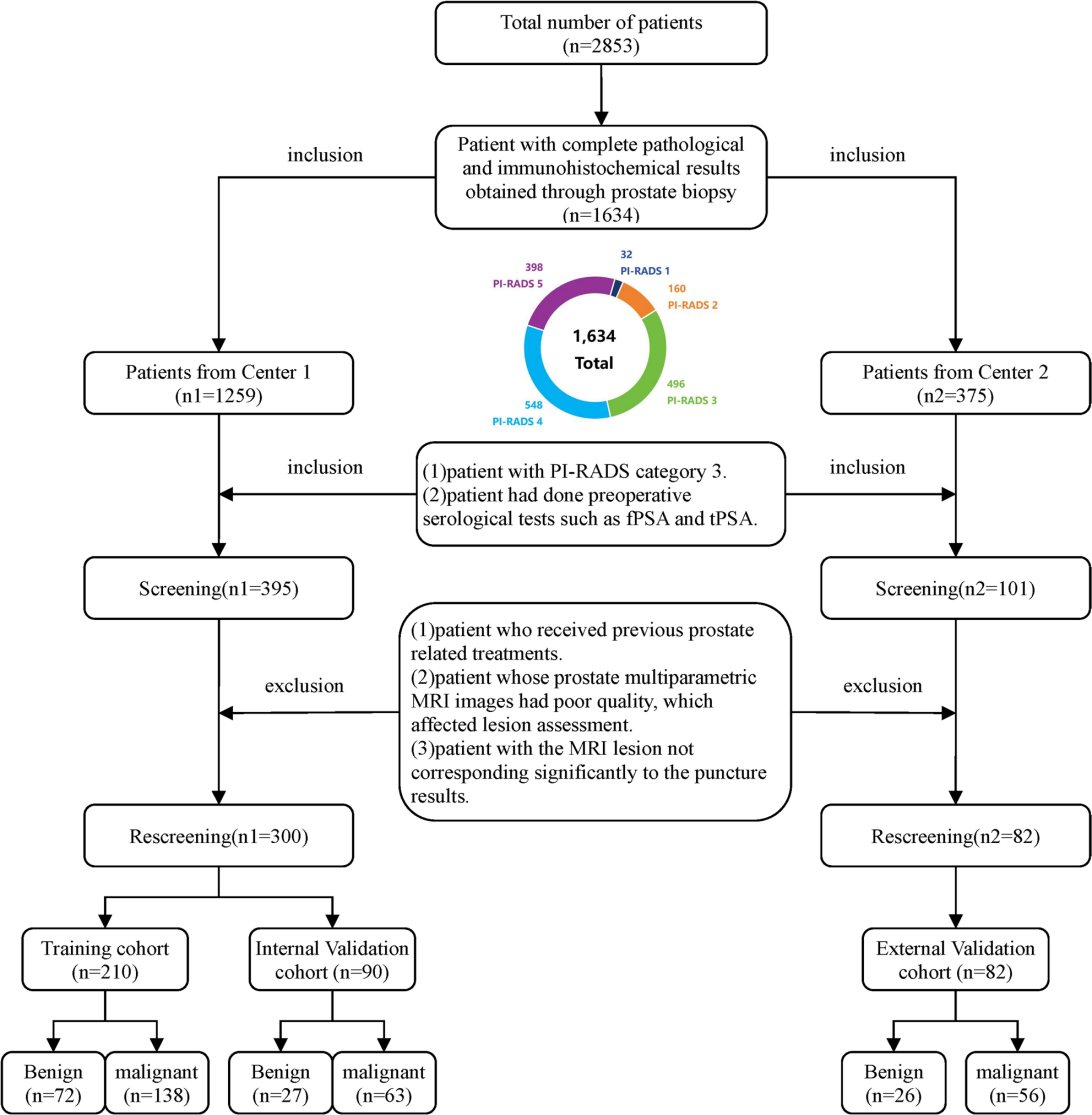
Enrollment process of the dual-center study population.

Clinical information and MRI imaging data from a total of 1,634 patients, who underwent multiparametric prostate MRI examinations between January 2022 and August 2024 at the two participating hospitals, were retrospectively analyzed. Inclusion criteria consisted of: (1) definitive biopsy outcomes confirmed by histopathology and immunohistochemistry; (2) prostate lesions classified as PI-RADS category 3 according to PI-RADS v2.1; and (3) availability of comprehensive preoperative serum markers, including fPSA and tPSA. Exclusion criteria included: (1) previous treatment involving prostate interventions, such as hormonal therapies, radiotherapy, or transurethral resection of the prostate prior to the current admission; (2) suboptimal MRI image quality hindering lesion assessment; and (3) biopsy findings not clearly matching identified MRI lesions. Following rigorous selection procedures (Fig. 1), the final sample consisted of 382 patients.

### MRI acquisition and image analysis

MRI scans were conducted utilizing Siemens MRI scanners (Germany), operating at field strengths of 1.5T and equipped with surface array coils. Patients were positioned supine, and the imaging protocol covered the entire prostate region comprehensively. Before scanning, patients were required to empty the bowel and moderately fill the bladder. MRI sequences included conventional transverse T2WI, DWI, and automatically generated ADC images. MRI parameters are detailed in Table 1.

**Table 1:** MRI scan parameters for the two centers.

Sequence	Parameters	Center 1 (SIEMENS 1.5T)	Center 2 (SIEMENS 1.5T)
T2WI	TR/TE, ms	3000,95	4110,95
	FOV, mm	190 × 100	200 × 200
	Thickness, mm	4.0	3.0
	Matrix	256 × 80	460 × 512
DWI	b value	0,1400	0,1000
	TR/TE, ms	6000,92	3430,90
	FOV, mm	230 × 90.6	256 × 256
	Thickness, mm	4.0	3.0
	Matrix	128 × 70	144 × 192

Two radiologists, blinded to pathological diagnoses and possessing 8 and 15 years of abdominal imaging experience, independently evaluated the MR images. Lesions were scored according to PI-RADS v2.1 standards. In case of discordance, a senior radiologist with 20 years of expertise reviewed the images and provided a definitive classification. For patients with multiple lesions, only the most clinically prominent lesion (defined as the lesion with the largest diameter) was selected for analysis to maintain statistical independence and avoid unit-of-analysis errors. After scoring, the following patient data were recorded: (1) basic patient information; (2) prostate volume (measured on axial T2-weighted images using the ellipsoid formula: length × width × height × 0.52, mL) and PSA density (PSAD = total PSA/prostate volume, ng/mL²);(3) tumor location (Peripheral Zone: 0; Transition Zone: 1; Involvement of Both Peripheral and Transition Zones: 2); (4) Apparent Diffusion Coefficient (ADCs, ×10^⁻6^ mm²/s); (5) lesion number (Single: 0; Multiple: 1); (6) lesion long diameter (Measured on the axial image at the largest cross-section of the largest nodule, mm); (7) lesion short diameter (Measured on the axial image at the largest cross-section of the largest nodule, mm); (8) lesion area (Long Diameter × Short Diameter, mm²); (9) lesion morphology(Round: 1; Oval: 2; Irregular: 3); (10) lesion margin (Well-Defined: 1; Ill-Defined: 2); (11) lesion position(Left: 1; Right: 2; Bilateral: 3).

### Pathological examination

Patients underwent either transrectal or transperineal prostate biopsy using a standard systematic 12-core sampling protocol. After formalin fixation, embedding, and sectioning, specimens were routinely examined using pathology and immunohistochemistry by a single pathologist. Patients were classified into benign (0, benign proliferative nodules) and malignant (1, PCa) groups.

### Deep learning process

#### Dataset preparation and image preprocessing

Data processing was performed using PyCharm Community Edition with Python 3.9. Rectangular ROIs containing PI-RADS 3 prostate lesions were manually delineated on the maximum cross-sectional images based on T2WI, DWI, and ADC sequences. All ROI images were then cropped to a standard size of 256 × 256 pixels. To minimize data heterogeneity, the transforms.Compose function integrated preprocessing steps, including normalization. To prepare MRI data for deep learning model input, pixel intensities of images were standardized using calculated mean and standard deviation values. The dataset from Center 1 was randomly partitioned into training and internal validation subsets at a 7:3 ratio for model development and preliminary evaluation, while the Center 2 dataset was exclusively utilized as an external validation group.

#### Convolutional neural network architecture

**Fig. 2:**
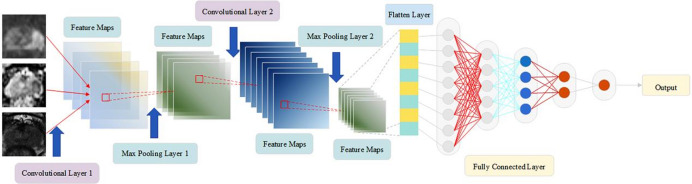
Flowchart of deep learning image processing. Cropped ROI images (input) undergo feature extraction via convolutional layers, generating multi-layer feature representations (blue rectangles). Dimensionality is subsequently reduced using pooling layers, and features are input into fully connected layers for transformation and output.

The model used a deep transfer learning framework based on ResNet101. Input MRI images were initially processed through convolutional layers. The resulting feature maps underwent dimensionality reduction via max-pooling layers. This convolutional-max pooling combination was repeated multiple times to extract deeper image features. The final feature maps were then flattened into one-dimensional vectors, which were fed into fully connected (FC) layers to derive high-level deep learning features (Fig. 2).

#### Model construction and feature selection

To prevent overfitting from redundant features, the mRMR and LASSO regression methods were employed. These techniques screened deep learning features, eliminating highly collinear variables. Significant features identified during the feature-selection stage were integrated into a logistic regression framework, which assigned weighted coefficients and established the final classification model.

**Fig. 3:**
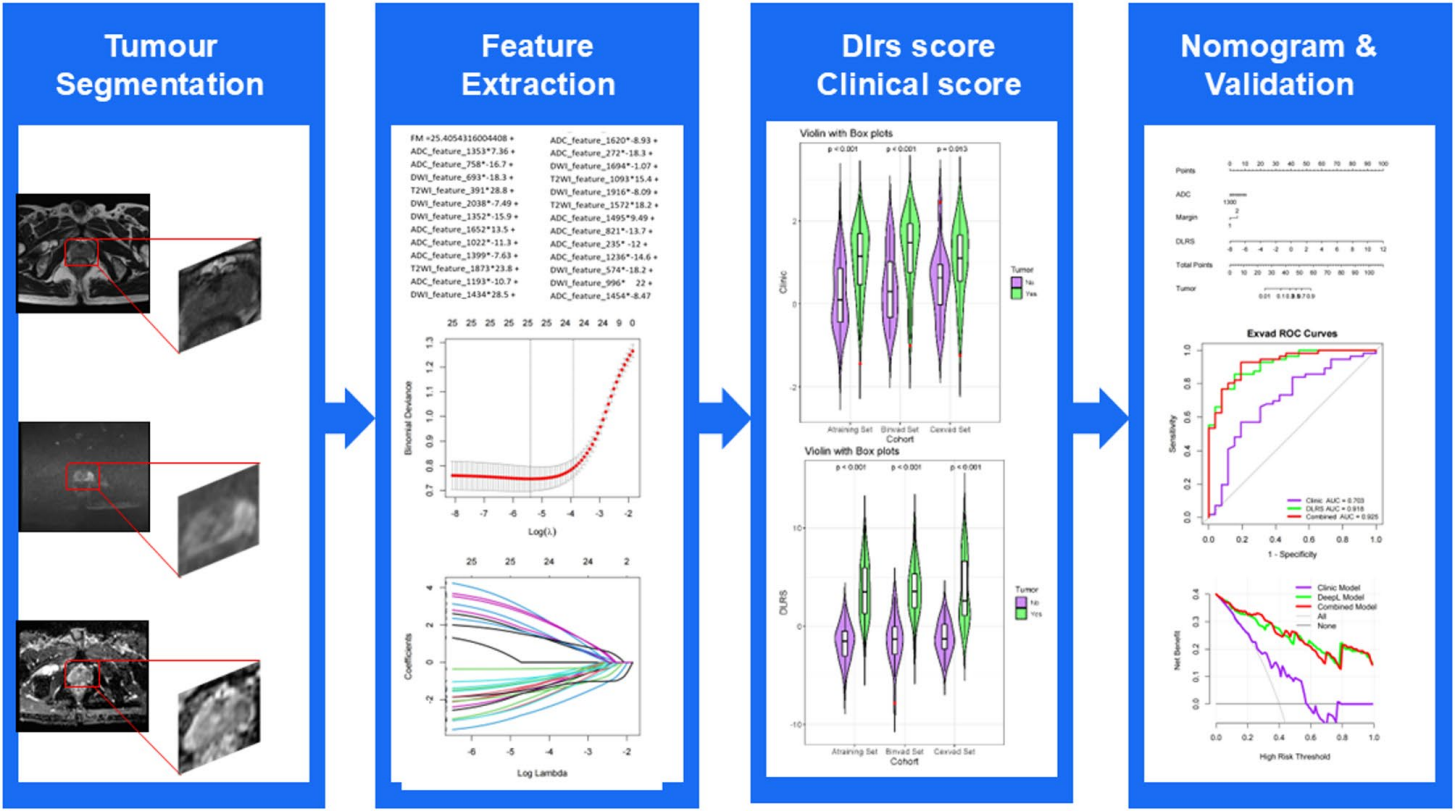
Flowchart illustrating construction of the DLRS model, clinical model, and combined deep learning nomogram.

Three distinct predictive models were developed: (1) a clinical-only model based purely on clinical data; (2) a deep learning-based radiomics signature (DLRS) model; and (3) an integrated model combining DLRS with clinical variables. Model performance across the training, internal validation, and external validation sets was assessed using metrics. Differences in model performance were statistically evaluated with DeLong’s test. To further enhance clinical practicality, the superior combined model was depicted visually as a nomogram. Calibration plots were created to verify consistency between predicted outcomes and actual clinical results. Decision curve analysis (DCA) quantified clinical utility at various threshold probabilities (Fig. 3).

### Statistical analysis

All statistical analyses were performed with R (version 3.6.1). Normality of data distribution was examined using the Kolmogorov–Smirnov test. Variables were reported as mean ± standard deviation (SD), or median and interquartile range (IQR). The Caret package was employed for dataset splitting and feature normalization. Accuracy, sensitivity, and specificity values were calculated from confusion matrices. Chi-square or Fisher’s exact tests analyzed categorical variables, and continuous variables were examined using independent-sample t-tests, one-way ANOVA, or appropriate nonparametric tests.

Both univariate and multivariate regression analyses were conducted to identify significant clinical and imaging predictors. ROC analysis was utilized to assess discriminative ability of each model, calculating AUC values along with corresponding 95% confidence intervals (CI). Model performance comparisons were statistically verified via the DeLong test. Calibration and decision curve analyses were executed with the rms and rmda packages, respectively. *p* < 0.05 indicated statistical significance.

## Results

### Patient characteristics

In total, 382 patients participated, with a median age of 72 years (IQR: 67.0–77.0). Statistical analysis showed no significant difference between the institutions regarding prostate cancer diagnosis rates (*p* = 0.825) (Table 2). Patients from Center 1 were randomly divided at a 7:3 ratio into training (*n* = 210) and internal validation groups (*n* = 90), while patients from Center 2 formed an external validation group (*n* = 82). Pathological analysis categorized patients into benign or malignant lesion groups, with the following distribution: in the training group, benign lesions were observed in 72 patients (34%) and malignant in 138 patients (66%); in the internal validation group, benign and malignant lesions were noted in 27 (30%) and 63 (70%) patients, respectively; and in the external validation cohort, 26 (31%) cases were benign and 56 (69%) were malignant.

In the training set, significant distinctions (*p* < 0.05) between benign and malignant lesions emerged in age, ADC values, total PSA (TPSA), lesion long-axis diameter, lesion location, lesion position, and lesion margin characteristics. However, no significant differences (*p* > 0.05) were found regarding free PSA (FPSA), short-axis diameter, lesion count, or lesion morphology. Within the internal validation group, significant differences (*p* < 0.05) between benign and malignant groups appeared across ADC value, age, TPSA, long-axis and short-axis diameters, lesion number, lesion position, location, morphology, and margin features, though FPSA was comparable between groups (*p* > 0.05). The external validation cohort displayed significant differences (*p* < 0.05) in ADC value, patient age, TPSA, lesion long-axis diameter, short-axis diameter, lesion position, and lesion location. Conversely, FPSA, lesion count, morphology, and margin characteristics showed no significant intergroup differences (*p* > 0.05) (Table 3).

**Table 2:** Baseline characteristics of the dual-center study population.

	Center 1(*n* = 300)	Center 2(*n* = 82)	*P*
Lesion			0.825
0	99(33.00%)	26(31.71%)	
1	201(67.00%)	56(68.29%)	
Location			0.064
0	109(36.33%)	30(36.59%)	
1	154(51.33%)	49(59.76%)	
2	37(12.33%)	3(3.66%)	
Number			0.63
0	269(89.67%)	75(91.46%)	
1	31(10.33%)	7(8.54%)	
Position			0.837
1	128(42.67%)	38(46.34%)	
2	105(35.00%)	27(32.93%)	
3	67(22.33%)	17(20.73%)	
Morphology			0.001*
1	18(6.00%)	15(18.29%)	
2	105(35.00%)	29(35.37%)	
3	177(59.00%)	38(46.34%)	
Margin			0.252
1	112(37.33%)	25(30.49%)	
2	188(62.67%)	57(69.51%)	
Age(y)	72.00(67.00,77.75)	71.00(66.75,76.25)	0.208
TPSA(µg/L)	10.24(6.72,16.85)	9.68(6.94,15.11)	0.602
FPSA(µg/L)	1.54(1.06,2.93)	1.51(0.98,2.48)	0.608
PSAD(ng/mL²)	0.19 (0.13,0.37)	0.20 (0.13,0.38)	0.738
LD(mm)	11.00(8.00,15.30)	10.65(7.00,14.00)	0.116
SD(mm)	8.00(5.00,10.82)	7.00(5.00,10.00)	0.29
ADC(×10⁻^6^ mm²/s)	716.11 ± 170.87	736.66 ± 167.91	0.333

**Table 3:** Comparison of clinical and imaging features of PI-RADS 3 nodules among the training, internal validation, and external validation cohorts.

Characteristic	Training		P1	in-valid		P2	Ex-valid		P3
	0 (*n* = 72)	1 (*n* = 138)		0 (*n* = 27)	1 (*n* = 63)		0 (*n* = 26)	1 (*n* = 56)	
ADC(×10⁻^6^ mm²/s	780.90 ± 161.40	697.22 ± 168.05	< 0.001	765.89 ± 138.82	662.10 ± 175.41	0.008	804.96 ± 178.38	704.95 ± 154.37	0.011
Age (y)	69.00 (66.00, 75.00)	73.00 (68.00, 78.00)	0.001	70.00(66.00, 72.50)	74.00 (69.00, 84.00)	< 0.001	69.00 (65.00, 72.00)	72.50 (67.00, 79.00)	0.016
TPSA (µg/L)	8.55 (6.59, 11.83)	11.77 (6.97, 19.94)	0.002	8.83 (5.67, 11.69)	13.24 (7.57, 19.56)	0.013	8.34 (6.15, 11.54)	10.99 (7.59, 18.73)	0.026
FPSA (µg/L)	1.52 (1.03, 2.06)	1.57 (1.16, 3.44)	0.118	1.57 (0.96, 2.28)	1.52 (0.90, 2.96)	0.663	1.41 (0.97, 2.37)	1.55 (1.02, 2.50)	0.594
PSAD(ng/mL²)	0.15(0.11,0.20)	0.19 (0.14,0.40)	< 0.001	0.15(0.11,0.23)	0.33 (0.18,0.63)	< 0.001	0.14(0.09,0.18)	0.28 (0.17,0.49)	< 0.001
LD(mm)	11.00 (7.00, 13.00)	12.00 (9.00, 16.00)	0.009	7.00 (6.00, 13.00)	15.00 (10.95, 22.00)	< 0.001	8.00 (6.00, 10.75)	12.00 (8.07, 16.00)	0.001
SD (mm)	7.50 (5.00, 9.25)	8.00 (5.00, 10.75)	0.169	5.00 (4.00, 8.50)	9.00 (7.65, 13.05)	< 0.001	5.50 (4.25, 7.75)	8.60 (5.78, 11.00)	0.003
Location			< 0.001			< 0.001			0.027
0	15 (20.83%)	61 (44.20%)		10 (37.04%)	23 (36.51%)		5 (19.23%)	25 (44.64%)	
1	57 (79.17%)	61 (44.20%)		17 (62.96%)	19 (30.16%)		21 (80.77%)	28 (50.00%)	
2	0 (0.00%)	16 (11.59%)		0 (0.00%)	21 (33.33%)		0 (0.00%)	3 (5.36%)	
Number			0.18			0.003			0.541
0	70 (97.22%)	126 (91.30%)		27 (100.00%)	46 (73.02%)		25 (96.15%)	50 (89.29%)	
1	2 (2.78%)	12 (8.70%)		0 (0.00%)	17 (26.98%)		1 (3.85%)	6 (10.71%)	
Position			0.002			< 0.001			0.028
1	35 (48.61%)	57 (41.30%)		11 (40.74%)	25 (39.68%)		16 (61.54%)	22 (39.29%)	
2	33 (45.83%)	46 (33.33%)		15 (55.56%)	11 (17.46%)		9 (34.62%)	18 (32.14%)	
3	4 (5.56%)	35 (25.36%)		1 (3.70%)	27 (42.86%)		1 (3.85%)	16 (28.57%)	
Morphology			0.062			< 0.001			0.346
1	7 (9.72%)	7 (5.07%)		3 (11.11%)	1 (1.59%)		6 (23.08%)	9 (16.07%)	
2	33 (45.83%)	47 (34.06%)		13 (48.15%)	12 (19.05%)		11 (42.31%)	18 (32.14%)	
3	32 (44.44%)	84 (60.87%)		11 (40.74%)	50 (79.37%)		9 (34.62%)	29 (51.79%)	
Margin			< 0.001			< 0.001			0.285
1	47 (65.28%)	38 (27.54%)		15 (55.56%)	12 (19.05%)		10 (38.46%)	15 (26.79%)	
2	25 (34.72%)	100 (72.46%)		12 (44.44%)	51 (80.95%)		16 (61.54%)	41 (73.21%)	

Note: Categorical variable codes — Lesion: 0 = Benign, 1 = Malignant; Location: 0 = Peripheral Zone, 1 = Transition Zone, 2 = Involvement of Both Peripheral and Transition Zones; Number: 0 = Single, 1 = Multiple; Position: 1 = Left, 2 = Right, 3 = Bilateral; Morphology: 1 = Round, 2 = Oval, 3 = Irregular; Margin: 1 = Well-Defined, 2 = Ill-Defined. Abbreviations: Age (y) = age in years; TPSA = Total Prostate-Specific Antigen (µg/L); FPSA = Free Prostate-Specific Antigen (µg/L); PSAD = Prostate-Specific Antigen Density (ng/mL²), calculated as total PSA divided by prostate volume, with prostate volume measured on axial T2-weighted images using the ellipsoid formula (length × width × height × 0.52, mL); LD = Long Diameter (mm), measured on the axial image at the largest cross-section of the target lesion; SD = Short Diameter (mm), measured on the axial image at the largest cross-section of the target lesion; ADC = Apparent Diffusion Coefficient (×10⁻⁶ mm²/s). Continuous variables with normal distribution are presented as mean ± standard deviation; non-normally distributed variables are presented as median (interquartile range); categorical variables are presented as n (%).

### Clinical model construction

**Fig. 4:**
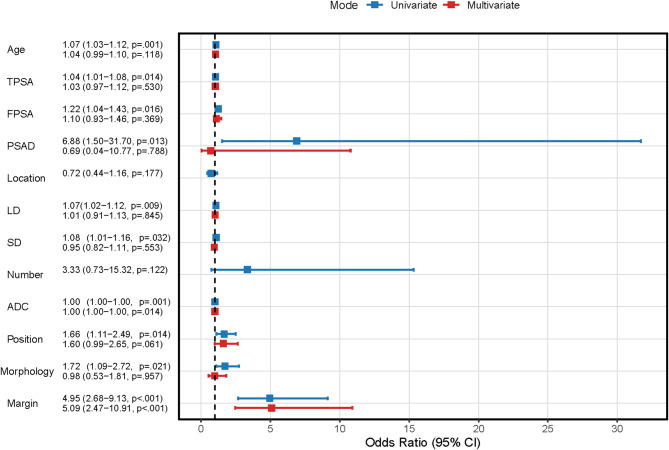
Univariate and multivariate regression analyses of clinical and radiological features.

Univariate logistic regression analyses within the training group highlighted significant distinctions (*p* < 0.05) between malignant and benign lesions regarding patient age, TPSA, FPSA, PSAD, lesion long-axis and short-axis diameters, ADC value, lesion side position, morphology, and margin features. Lesion location and number showed no statistical significance. Multivariate logistic regression subsequently identified ADC value (OR = 1.00, *p* = 0.014) and lesion margin features (OR = 5.09, *p* < 0.001) as independent predictive factors differentiating malignant from benign PI-RADS 3 nodules (Fig. 4). A clinical predictive model was established using these variables through logistic regression.

**Fig. 5:**
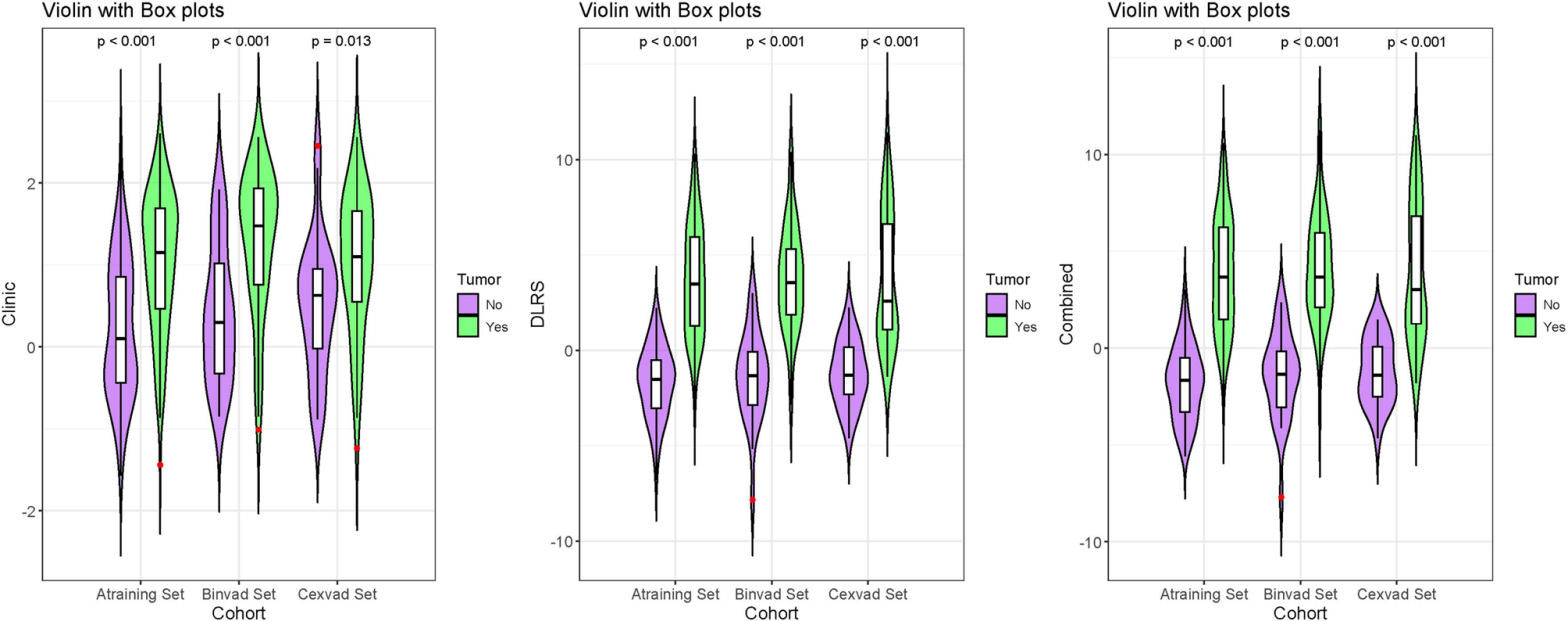
Violin plots representing clinical, DLRS, and combined models. The horizontal line represents the median; black vertical lines represent ranges from smallest to largest non-outliers.

**Fig. 6:**
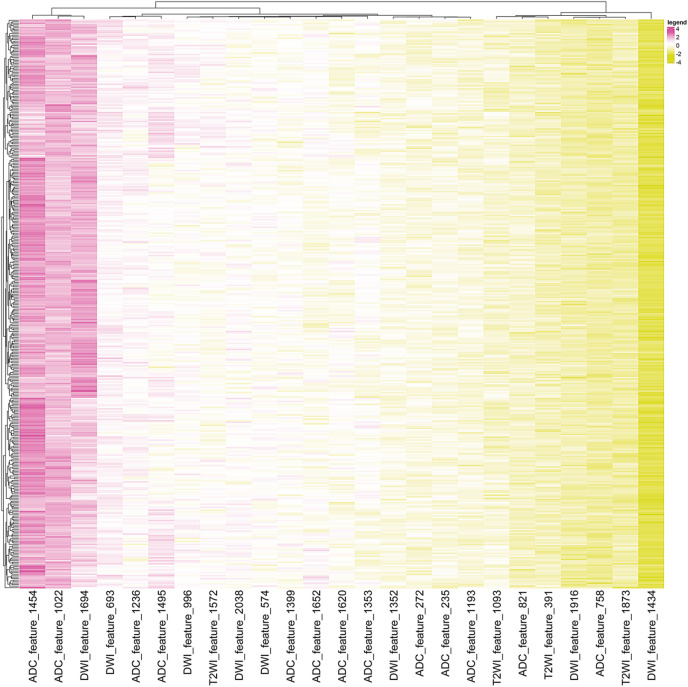
Heat map illustrating correlations of radiomics features used in the DLRS model. Horizontal axis represents image features.

The violin and box plots showed that the radiomics and clinical scores in the malignant group were higher across all study cohorts (Fig. 5). Through LASSO regression, 6119 redundant features were removed from 6144 deep learning features derived from T2WI, DWI, and ADC sequences. Ultimately, 25 key features were retained for model construction: 13 from ADC sequences, 8 from DWI sequences, and 4 from T2WI sequences. Heat map results indicated that ADC_feature_1454, ADC_feature_1022, and DWI_feature_1694 had high feature weights and strong discriminative capability in the model (Fig. 6).

DLRS = 25.4054316004408 + ADC_feature_1353*7.36 + ADC_feature_758*−16.7 + DWI_feature_693*−18.3 + T2WI_feature_391*28.8 + DWI_feature_2038*−7.49 + DWI_feature_1352*−15.9 + ADC_feature_1652*13.5 + ADC_feature_1022*−11.3 + ADC_feature_1399*−7.63 + T2WI_feature_1873*23.8 + ADC_feature_1193*−10.7 + DWI_feature_1434*28.5 + ADC_feature_1620*−8.93 + ADC_feature_272*−18.3 + DWI_feature_1694*−1.07 + T2WI_feature_1093*15.4 + DWI_feature_1916*−8.09 + T2WI_feature_1572*18.2 + ADC_feature_1495*9.49 + ADC_feature_821*−13.7 + ADC_feature_235* −12 + ADC_feature_1236*−14.6 + DWI_feature_574*−18.2 + DWI_feature_996* 22 + ADC_feature_1454*−8.47.

### Construction and evaluation of the combined model

**Fig. 7:**
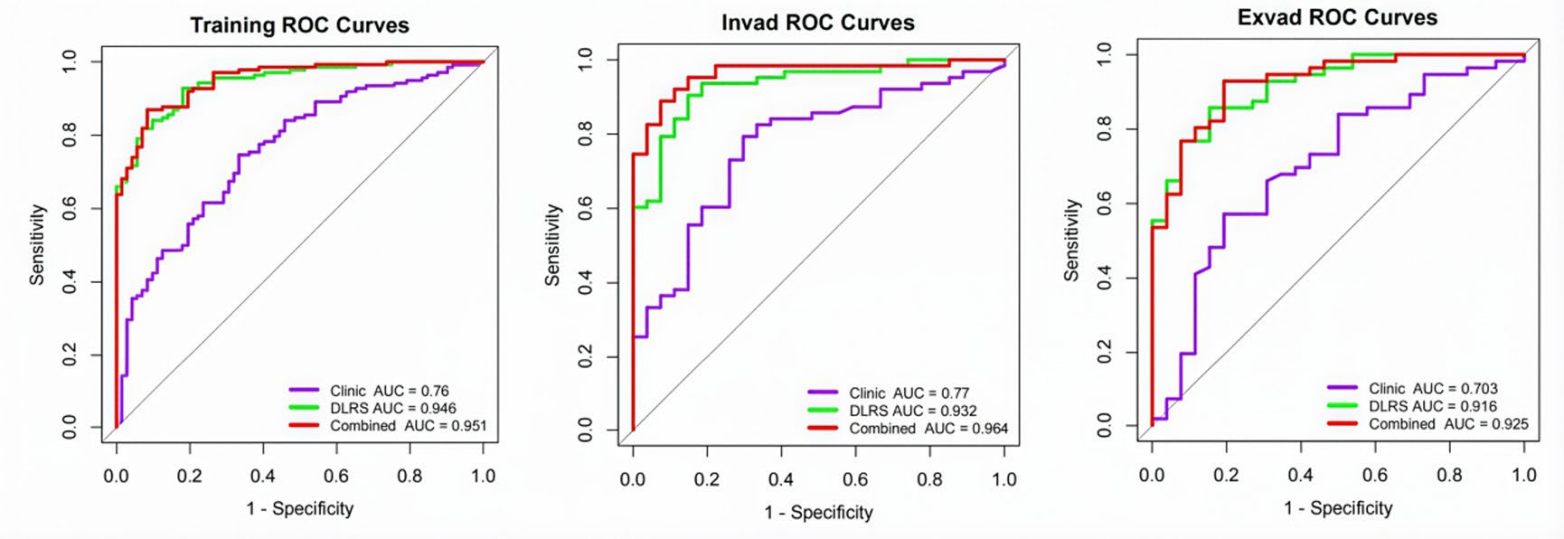
ROC analysis of predictive models in the training, internal validation, and external validation cohorts.

Subsequently, a combined predictive model was created by integrating clinical variables and DLRS data. ROC analyses for clinical, DLRS, and combined predictive models across training, internal, and external validation sets are illustrated in Fig. 7. The clinical model achieved AUC values of 0.760, 0.770, and 0.703 in the respective cohorts. The DLRS model demonstrated enhanced performance, yielding AUC values of 0.946, 0.932, and 0.918, respectively, while the combined model exhibited the highest predictive capability with AUCs of 0.951, 0.964, and 0.925 across cohorts (Table 4).

**Table 4:** ROC analysis results for the three models.

Cohort	MODEL	AUC(95%CL)	Sensitivity	Specifcity	Accuracy	PPV	NPV
Training	Clinical	0.76(0.692–0.827)	0.746	0.667	0.719	0.811	0.578
	DLRS	0.946(0.918–0.973)	0.819	0.931	0.857	0.958	0.728
	Comblined	0.951(0.925–0.977)	0.87	0.917	0.886	0.952	0.786
Internal validation	Clinical	0.77(0.666–0.874)	0.794	0.704	0.767	0.862	0.594
	DLRS	0.932(0.879–0.984)	0.905	0.852	0.889	0.934	0.793
	Comblined	0.964(0.928–1)	0.889	0.926	0.900	0.966	0.781
External validation	Clinical	0.703(0.577–0.829)	0.571	0.808	0.646	0.865	0.467
	DLRS	0.918(0.86–0.976)	0.857	0.846	0.854	0.923	0.733
	Comblined	0.925(0.868–0.983)	0.929	0.808	0.890	0.912	0.840

The DeLong test comparisons (Table 5) indicated that both DLRS and combined models surpassed the clinical model in predictive accuracy across all cohorts (*p* < 0.05). Nevertheless, there was no significant difference (*p* > 0.05) between the DLRS and combined models. In conclusion, the clinical model displayed lower diagnostic accuracy than both DLRS and combined models, with the combined model showing equivalent accuracy to the DLRS model.

**Table 5:** Delong test results comparing the three models.

Model	Training	Internal validation	External validation
Clinic v DLRS	<0.001	0.009	0.002
Clinic v Combined	<0.001	<0.001	0.001
DLRS v Combined	0.307	0.104	0.574

### Nomogram construction and evaluation

**Fig. 8:**
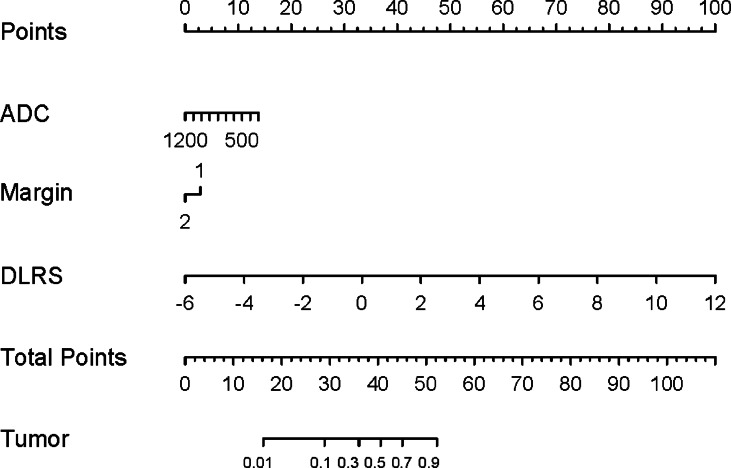
Nomogram of the combined model.

**Fig. 9:**
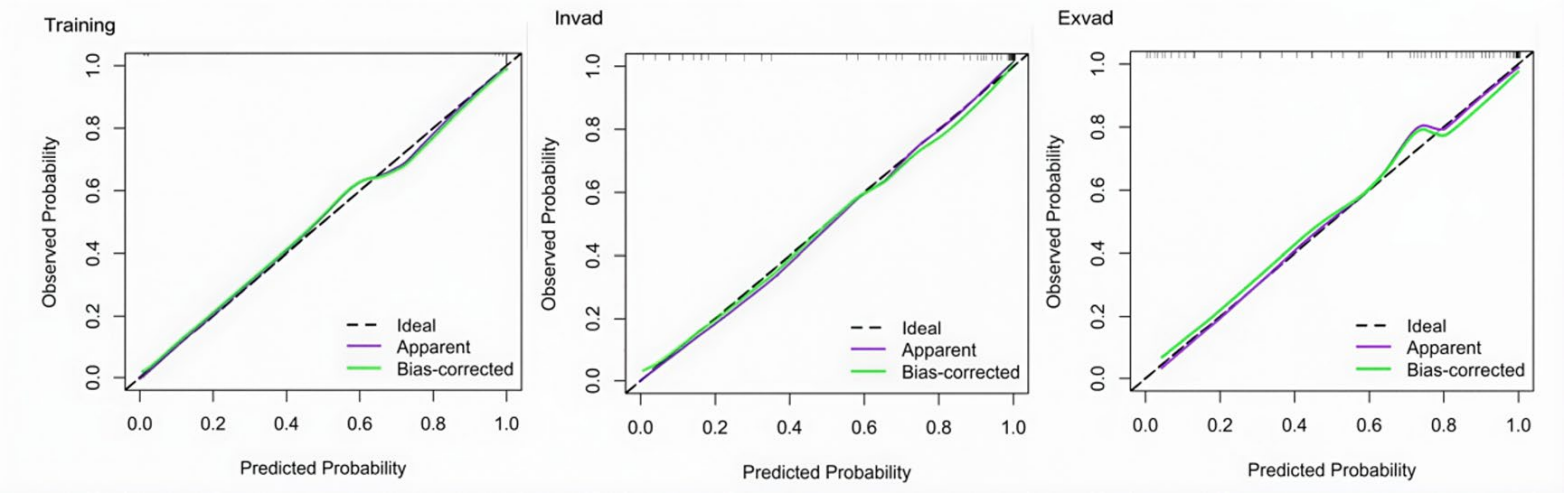
Calibration curves for the training, internal validation, and external validation cohorts.

**Fig. 10:**
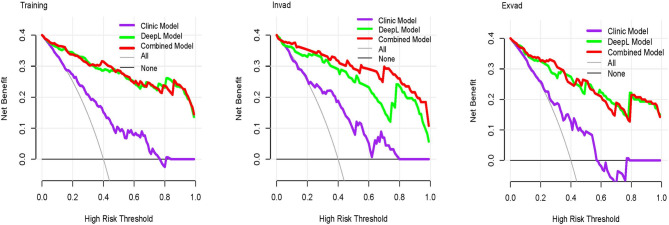
DCA was conducted to evaluate the clinical utility of the clinical, DLRS, and combined models in training, internal validation, and external validation cohorts.

A nomogram derived from the combined model was established for clinical application (Fig. 8). Calibration curves illustrated strong concordance between the predicted and actual pathological outcomes, confirming effective model calibration (Figs. 9). Furthermore, DCA demonstrated that the nomogram offered substantial clinical benefit across a wide spectrum of threshold probabilities, underscoring its practical value for clinical decision-making (Figs. 10).

## Discussion

This research established and assessed three separate predictive frameworks, a clinical-only model, a DLRS model, and an integrated model combining both clinical indicators and multiparametric MRI features, using data from PI-RADS 3 prostate lesions acquired at two distinct medical centers. The main goal was to effectively classify lesions as benign or malignant. Findings revealed that both the DLRS and integrated models exhibited superior diagnostic performance compared to the traditional clinical model across training, internal validation, and external validation groups, emphasizing their enhanced preoperative predictive accuracy. Additionally, the combined-model-based nomogram provided a practical and individualized preoperative risk assessment tool with strong clinical applicability.

The conventional clinical model, reliant solely on routine MRI imaging features, produced AUC results of 0.760, 0.770, and 0.703 for the training, internal validation, and external validation groups, respectively. Nevertheless, these performance measures were significantly inferior when compared to those generated by the DLRS and integrated models. According to PI-RADS v2.1 standards, PI-RADS category 3 lesions remain equivocal; thus, clinical decision-making generally necessitates additional clinical considerations and evaluations^20^. Although three experienced radiologists independently evaluated the images, improving diagnostic reliability to some extent, traditional imaging methods remain limited in accurately assessing PI-RADS 3 lesions. Therefore, more accurate and efficient preoperative prediction methods are urgently required.

Both univariate and multivariate logistic regression evaluations identified ADC measurements and lesion margin characteristics as independent factors predictive of benign versus malignant status in PI-RADS 3 prostate lesions. ADC, as a quantitative imaging biomarker derived from DWI, reflects water molecule diffusion within tissues. It indirectly reveals the pathological features and tumor differentiation degree, correlating significantly with Gleason score^21^. The heatmap demonstrated that the ADC sequence had the largest number of relevant features, with ADC_feature_1454 and ADC_feature_1022 exhibiting the highest expression levels and weights. This finding aligns with previous studies^22,23^, confirming ADC values as independent predictors of clinically significant PCa (csPCa). The correlation between lesion margin characteristics and malignancy is consistent with findings by BoschHeidgen et al.^24^. This relationship might be attributed to the invasive growth pattern of malignant tumor cells. Imaging studies of primary liver cancer have similarly identified non-smooth tumor margins as biomarkers indicating invasive tumor growth and surrounding tissue infiltration^25,26^. Consequently, blurred edges and indistinct lesion boundaries observed in PCa imaging likely represent shared biological markers among various malignant tumors. These subtle imaging features are challenging for human visual assessment; however, radiomics can quantitatively extract and analyze high-dimensional image data, thereby revealing tumor heterogeneity^27^.

Since Gleason score 6 prostate cancer is pathologically classified as low-grade, well-differentiated, slow-growing, and minimally aggressive, most previous studies have primarily focused on clinically significant prostate cancer, defined as Gleason score > 6. However, clinical evidence indicates that biopsy-based Gleason scores are often lower than those determined from postoperative histopathology^28^. When Gleason score upgrading occurs in prostate cancer patients, it is associated with increased biochemical recurrence rates and reduced cancer-specific survival, significantly impacting prognosis^29^. To account for the potential risk posed by these more aggressive phenotypes that are initially undetected by biopsy, as well as their corresponding influence on outcomes, we have included Gleason score 6 tumors in the defined malignant category.This study utilized the T2WI, DWI, and ADC sequences of mpMRI to construct a deep learning model that effectively predicted benign versus malignant PI-RADS 3 prostate nodules. Compared with traditional machine learning, which relies on precise manual lesion segmentation, the DLRS method automatically extracts high-dimensional features from raw images. This approach captures complex correlations between features and maximizes representation of lesion heterogeneity. A single-slice annotation strategy was employed, maintaining prediction accuracy while significantly reducing labor and time. Moreover, this method avoids extensive manual delineation and feature bias caused by human intervention, facilitating comprehensive extraction of disease-related radiomics information. Unlike previous studies focused solely on machine learning, this research combined deep learning with clinical variables, constructing three models validated on internal and external cohorts. DeLong test results revealed that the DLRS model exhibited excellent predictive performance. Thus, the DLRS model demonstrated strong generalizability suitable for clinical diagnostics.

Earlier research employing MRI-based radiomics approaches has similarly targeted the identification of malignancy within PI-RADS 3 lesions. For instance, Hou et al.^30^ previously created radiomics models combining imaging traits from T2WI, ADC, and DWI sequences, achieving AUC results of 0.744 in training cohorts and 0.738 in validation cohorts. Their integrated radiomics strategy surpassed models using single imaging sequences alone in detecting clinically significant PCa. Similarly, Hectors et al.^19^ developed a random forest classifier exclusively utilizing radiomic data from T2WI, which yielded promising predictive outcomes (AUC = 0.76, *P* = 0.022) within their testing group. Despite their merits, these studies faced constraints such as relatively limited sample sizes and dependence on singular imaging modalities. Conversely, our study employed deep learning methodologies to extract comprehensive image characteristics, subsequently combining quantitative information with potential biological underpinnings to develop an integrated diagnostic model. This comprehensive method achieved notable AUC scores of 0.964 and 0.925 in internal and external validation cohorts, respectively, demonstrating significant reliability, generalization potential, and practicality as a non-invasive diagnostic tool for discerning benign from malignant PI-RADS 3 lesions. Calibration curves for the combined-model-based nomogram showed predictions highly consistent with postoperative pathology. DCA further confirmed its significant clinical applicability, underscoring the model’s reliability and practical value. Thus, the nomogram developed from the combined model provides a straightforward, efficient preoperative risk assessment, potentially guiding clinicians in personalized diagnosis and treatment planning.

Nevertheless, several limitations of this study should be recognized. First, inherent selection bias cannot be completely excluded given the retrospective design. Second, pathology outcomes were established by standard systematic 12-core biopsy rather than radical prostatectomy, which raises the possibility of incomplete lesion correspondence between biopsies and MRI findings. Future studies should adopt MRI-targeted fusion biopsy where feasible to enhance diagnostic accuracy. Third, the maximum b-value for diffusion-weighted imaging at Center 2 was 1000 s/mm², falling below the currently recommended threshold of ≥ 1400 s/mm² in prostate MRI guidelines. This relatively lower b-value may reduce the sensitivity of DWI for detecting clinically significant lesions and could compromise the accuracy and reliability of ADC measurements, potentially affecting the diagnostic performance of the model. Fourth, the pathological endpoint in our study was defined as a binary classification of any-grade prostate cancer versus non-cancer, rather than a comparison between clinically significant prostate cancer (csPCa) and non-significant disease. This definition may have attenuated the independent predictive value of certain clinical indicators such as PSAD; future studies are encouraged to adopt csPCa as the pathological endpoint to better elucidate the clinical relevance of these indicators in the target population. Although external validation was performed, subsequent research should include larger, prospective multicenter cohorts with standardized DWI acquisition protocols across diverse imaging equipment to further verify the clinical applicability and broad generalizability of the proposed models.

In conclusion, both the DLRS and the integrated deep learning models derived from multiparametric MRI exhibited high accuracy in distinguishing benign from malignant prostate lesions categorized as PI-RADS 3, confirmed by validation across two institutions. The derived nomogram from the integrated model also presented notable clinical value, potentially serving as an important non-invasive tool to guide individualized diagnostic and treatment strategies in managing PCa.

## Supplementary Information

Below is the link to the electronic supplementary material.

Supplementary Material 1

Supplementary Material 2

## Data Availability

The datasets used and/or analyzed during the current study are available from the corresponding author on reasonable request.

## Abbreviations

AUC: Area under the curve
CsPCa: Clinically significant prostate cancer
CNN: Convolutional Neural Network
DCA: Decision curve analysis
CI: Confidence interval
DWI: Diffusion-weighted imaging
Ex-valid: External validation
FPSA: Free prostate specific antigen
In-valid: Internal validation
LASSO: Least absolute shrinkage and selection order
LD: Long diameter
mRMR: Maximum relevance minimum redundancy
MpMRI: multiparametric MRI
PCa: Prostate cancer
PI-RADS: Prostate imaging reporting and data system
ROC: Receiver operating characteristic
ROI: Region of Interest
SD: Short diameter
T2WI: T2-weighted imaging
TPSA: Total prostate specific antigen
