# Supplementary material for: Deep learning based on multiparametric MRI for differentiating benign from malignant PI-RADS 3 prostate lesions: a dual-center study
Source: Sci Rep. 2026 Jun 5;16:25757. doi: 10.1038/s41598-026-56643-x (PMC13482369; doi:10.1038/s41598-026-56643-x)
Supplement: Supplementary file 1 [file 41598_2026_56643_MOESM1_ESM.docx]

**Funding**

This work was supported by the Project of Taizhou Science and Technology Bureau in Zhejiang Province（25ywa29).
