# Supplementary material for: Deep learning based on multiparametric MRI for differentiating benign from malignant PI-RADS 3 prostate lesions: a dual-center study
Source: Sci Rep. 2026 Jun 5;16:25757. doi: 10.1038/s41598-026-56643-x (PMC13482369; doi:10.1038/s41598-026-56643-x)
Supplement: Supplementary file 2 [file 41598_2026_56643_MOESM2_ESM.docx]

**Statement on the Waiver of Informed Consent**

This retrospective study was reviewed and approved by the ethics committees of both participating institutions. The Medical Ethics Committee of Taizhou First People's Hospital (Approval No. 2024-KY006-01) and the Medical Ethics Committee of Putuo District People's Hospital, Shanghai (Approval No. 2022-07) approved the study and granted a waiver of informed consent given the retrospective nature of the study and the use of anonymized patient data.
